# The beneficial effect of baricitinib on ultrasound-detected synovial inflammation and bone damage in rheumatoid arthritis: Preliminarily data from single center-based observational study for 24 weeks

**DOI:** 10.1097/MD.0000000000026739

**Published:** 2021-07-30

**Authors:** Seong-Kyu Kim, Ui Hong Jung, Ji-Won Kim, Jung-Yoon Choe

**Affiliations:** Division of Rheumatology, Department of Internal Medicine, Catholic University of Daegu School of Medicine, Daegu, Republic of Korea.

**Keywords:** baricitinib, disease activity, rheumatoid arthritis, synovitis, ultrasound

## Abstract

Baricitinib is a Janus kinase (JAK) inhibitor that selectively blocks against JAK1 and JAK2 signaling. This study aimed to determine the effect of baricitinib on disease activity based on musculoskeletal ultrasound in patients with rheumatoid arthritis (RA).

A total of 20 patients with RA receiving baricitinib for 24 weeks were assessed. Ultrasound scores of gray scale and power Doppler synovitis, joint effusion, and bone erosion in each patient were assessed between baseline and 24 weeks for 27 affected joints. Disease activity in RA was evaluated using the disease activity score for 28-joint count with erythrocyte sediment rate (DAS28-ESR), simplified disease activity index (SDAI), and clinical disease activity index (CDAI).

Treatment with baricitinib for 12 weeks and 24 weeks significantly decreased disease activity composites such as DAS28-ESR, SDAI, and CDAI (*P* < .001 for all). Treatment with baricitinib for 24 weeks improved ultrasound-detected gray-scale and power Doppler synovitis and joint effusion compared to baseline (*P* = .002, *P* = .030, and *P* = .002, respectively). Bone erosion scores were not different between baseline and 24 weeks (*P* = .317). There were no differences in ultrasound abnormalities for improvement based on DAS28-ESR. Changes in power Doppler score were significantly associated with changes in DAS28-ESR (β = 0.590, *P* = .044), but not SDAI and CDAI.

This study demonstrates that baricitinib treatment has a favorable effect on ultrasound-detected abnormalities including synovitis and bone erosion in patients with RA.

## 1 Introduction

Rheumatoid arthritis (RA) is a complex autoimmune disease characterized by involvement of articular and extra-articular structures that ultimately leads to functional disability and poor quality of life.^[1]^ The main target of RA treatment is to improve clinical symptoms and signs, suppress inflammatory changes within affected joints, and prevent structural joint damage. Reliable clinical measures have been developed to assess arthritis in clinical trials and daily practice, such as the disease activity score in 28 joints erythrocyte sedimentation rate (DAS28-ESR), simplified disease activity index (SDAI), and clinical disease activity index (CDAI).^[2]^ Furthermore, musculoskeletal ultrasound is an important imaging tool used to assess disease activity and monitor treatment response.^[3]^ Ultrasound has been recognized as an imaging biomarker to evaluate treatment monitoring in patients with RA treated with biological disease-modifying antirheumatic drugs (bDMARDs) or targeted synthetic DMARDs.^[4,5,6]^

Baricitinib is a small-molecule Janus kinase (JAK) inhibitor that selectively suppresses intracellular cell signaling against JAK1 and JAK2, but has weak activity against JAK3.^[7]^ It was developed to treat patients with refractory RA who do not respond to conventional DMARDs. Extensive evidence has demonstrated that baricitinib has safety and clinical efficacy in managing patients with RA.^[8,9,10,11]^ Several studies demonstrated that baricitinib blocked structural joint damage through significant inhibition of radiographic progression in patients with RA and clinical improvement.^[9,12,13]^ Ultrasound findings after short-term baricitinib treatment for 3 months were associated with clinical disease activity markers.^[14]^ However, there is still a lack of data using ultrasound to evaluate treatment response to baricitinib. The aim of this study is to assess the clinical usefulness of ultrasound in monitoring disease activity in patients with RA treated with baricitinib for 24 weeks.

## 2 Materials and methods

### 2.1 Study populations

This study included 20 patients who met the 1987 American College of Rheumatology revised classification criteria for RA and were enrolled from September 2019 to July 2020.^[15]^ Subjects who participated in the study were patients who started baricitinib treatment due to insufficient response to treatment with conventional synthetic disease modifying antirheumatic drugs (csDMARDs) and/or bDMARDs. All patients received baricitinib 4 mg for 24 weeks and completed ultrasound examinations both at baseline and at 24 weeks were included in the study. This study was approved by the Institutional Review Board at Daegu Catholic University Medical Center (CR-20-154-L).

### 2.2 Collection of clinical information

Clinical data at baseline included age, gender, and disease duration at study enrollment. At the first administration of baricitinib, other medications used simultaneously were identified, such as methotrexate, corticosteroids, and nonsteroidal anti-inflammatory drugs.

The disease activity of patients with RA was assessed at both baseline and at 24 weeks after enrollment. Markers for disease activity included tender joint count (TJC), swollen joint count (SJC), patients global visual analog scale (VAS, mm), physician VAS, C-reactive protein (mg/L), and erythrocyte sediment rate (ESR, mm/h). After using these individual parameters, disease activity indexes including the DAS28-ESR, SDAI, and CDAI were calculated using the following formulas: DAS28 = 0.56 × √(TJC28) + 0.28 × √(SJC28) + 0.70 × ln (ESR) + 0.014 × patient global VAS (0–100 mm), SDAI = TJC28 + SJC28 + CRP (mg/L)/10 + patient global VAS (0– 100 mm)/10 + physician global VAS (0–100 mm)/10, and CDAI = TJC28 + SJC28 + patient global VAS (0–100 mm)/10 + physician global VAS (0–100 mm)/10.

### 2.3 Ultrasound assessment

**Figure 1:**
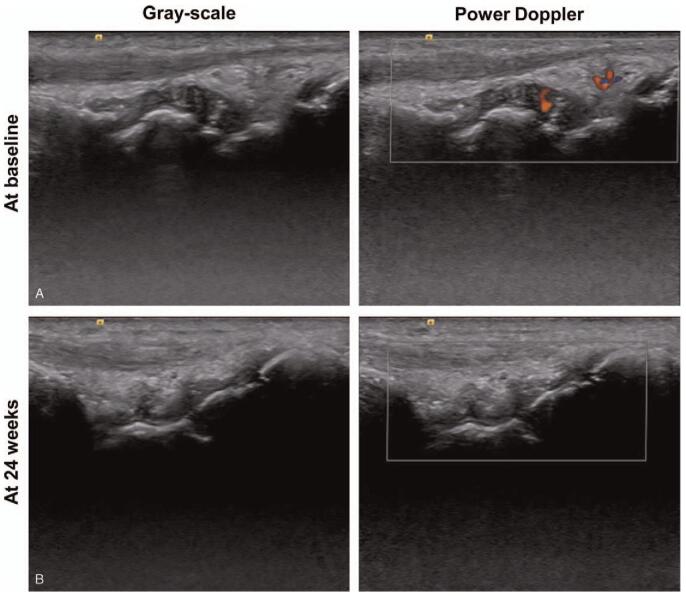
Ultrasound images at baseline and 24 wk after baricitinib treatment in rheumatoid arthritis. Representative figure for gray-scale and power Doppler ultrasound images of wrist joint in a patient with rheumatoid arthritis. (A) Gray-scale synovial hypertrophy and joint effusion with widening of joint space and Doppler synovitis was also noted at baseline. (B) Gray-scale synovial hypertrophy and joint effusion within joint space was decreased and power Doppler signal was disappeared after treatment with baricitinib 4 mg for 24 wk.

Ultrasound-based joint assessment was conducted by evaluating 1 or 2 of the most painful joints at baseline and then reassessing the same affected joint after 24 weeks of baricitinib treatment (Fig. 1). One rheumatologist (June UH) who completed a training program provided by the Korean College of Rheumatology performed ultrasound examinations. Ultrasound examinations used the ACUSON S2000 Ultrasound System (Siemens Healthineers, Seoul, Korea) with a 5 to 14 MHz linear transducer (14L5).

The 27 joints included in the joint ultrasound assessment were knee (n = 4), elbow (n = 4), foot (n = 1), shoulder (n = 10), wrist (n = 5), and hand joints (n = 3). Ultrasound was performed at standardized joint positions and a probe application was used for each joint. Ultrasound scoring of affected joints for joint effusion, gray-scale synovial hypertrophy, power Doppler synovitis, and bone erosion was performed according to the definition provided by Szkudlarek et al (Supplemental Digital Content Table 1).^[16]^

Intraclass correlation coefficient with 95% confidential interval (CI) for intraobserver reliability was calculated; 0.945 (95% CI 0.846–0.981) for joint effusion, 0.832 (95% CI 0.571–0.940) for gray-scale synovial hypertrophy, 0.822 (95% CI 0.549–0.936) for power Doppler synovitis, 0.896 (95% CI 0.719–0.964) for bone erosion.

### 2.4 Statistical analysis

Data were described as number with percentage (%) for nominal variables and median with interquartile range (IQR) for continuous variables. The difference in ultrasound findings such as joint effusion, gray-scale synovitis, power Doppler synovitis, and bone erosion between baseline and 24 weeks was assessed by Wilcoxon signed rank test. The differences in changes of ultrasound findings among 3 treatment response groups (good, moderate, and no improvement based on DAS28-ESR) were assessed by Kruskal–Wallis test. Correlation between changes of DAS28-ESR and ultrasound findings at 24 weeks was calculated using multivariate regression analysis after adjusting confounding factors. Statistical significance was considered to be *P* < .05. Statistical analyses were performed using IBM SPSS Statistics 19.0 (IBM Corp., Armonk, NY).

## 3 Results

### 3.1 Baseline characteristics of enrolled patients

A total of 20 patients who were treated with baricitinib for 24 weeks was enrolled in this study (Table 1). The median age of patients was 53.5 years (IQR 47.5 – 61.3). Most patients were female (n = 15, 75.0%). The median disease duration was 85.0 months (IQR 31.5 – 158.8). Disease activity markers including SJC, TJC, patient VAS, physician VAS, ESR, and CRP are illustrated in Table 1. Five patients who failed to achieve clinical response by bDMARDs and 15 patients who were not exposed to any bDMARDs were included in this study. Twenty-seven joints in 20 patients were assessed in this study, including knee (n = 4), elbow (n = 4), foot (n = 1), shoulder (n = 10), wrist (n = 5), and hand joints (n = 3).

**Table 1:** Baseline characteristics in enrolled patients (n = 20).

Variables	Results
Age (yr)	53.5 (47.5–61.3)
Sex, female (n, %)	15 (75.0)
Disease duration (mo)	85.0 (31.5–158.8)
Hypertension (n, %)	3 (15.0)
Diabetes mellitus (n, %)	1 (5.0)
RF positivity (n, %)	15 (75.0)
Anti-CCP antibody positivity (n, %)	17 (85.0)
Disease activity markers	
Swollen joint count	4.0 (2.0–6.5)
Tender joint count	7.0 (2.3–10.8)
Patient VAS	70.0 (60.0–80.0)
Physician VAS	60.0 (50.5–66.3)
ESR (mm/h)	33.5 (24.3–64.8)
CRP (mg/L)	16.3 (4.3–31.9)
csDMARDs (n, %)	
Methotrexate	20 (100.0)
Hydrochloroquine	2 (10.0)
Sulfasalazine	12 (60.0)
Leflunomide	8 (40.0)
Tacrolimus	2 (10.0)
bDMARDs (n, %)^∗^	5 (25.0)
Corticosteroid (n, %)	19 (95.0)
Corticosteroid (mg/d)	5.0 (2.5–5.0)
NSAIDs (n, %)	20 (100.0)
Joint assessment (n = 27)	
Knee	4 (14.8)
Elbow	4 (14.8)
Foot	1 (3.7)
Shoulder	10 (37.0)
Wrist	5 (18.5)
Hand	3 (11.1)

### 3.2 Clinical response to baricitinib treatment for 24 weeks

Treatment with baricitinib markedly improved disease activity assessed by DAS28-ESR, SDAI, and CDAI (Table 2). DAS28-ESR, SDAI, and CDAI at 12 weeks significantly improved compared to baseline (*P* < .001 for all). At 24 weeks, DAS28-ESR, SDAI, and CDAI were even more improved compared to baseline (*P* < .001 for all). Treatment response based on DAS28-ESR was good for 13 patients, moderate for 4 patients, and no improvement for 3 patients.

**Table 2:** Changes of clinical response after baricitinib treatment for 12 and 24 wk.

	Baseline	12 weeks	*P*-value^∗^	24 weeks	*P*-value^∗^
DAS28-ESR	5.62 (4.50–5.96)	3.89 (2.75–4.21)	*P* < .001	2.95 (2.30–3.90)	*P* < .001
SDAI	24.2 (19.3– 30.8)	8.75 (6.85–12.22)	*P* < .001	4.93 (3.19–8.10)	*P* < .001
CDAI	22.3 (17.9–30.4)	8.60 (6.70–12.00)	*P* < .001	4.80 (2.95–7.83)	*P* < .001

### 3.3 Ultrasound findings after treatment with baricitinib for 24 weeks

**Figure 2:**
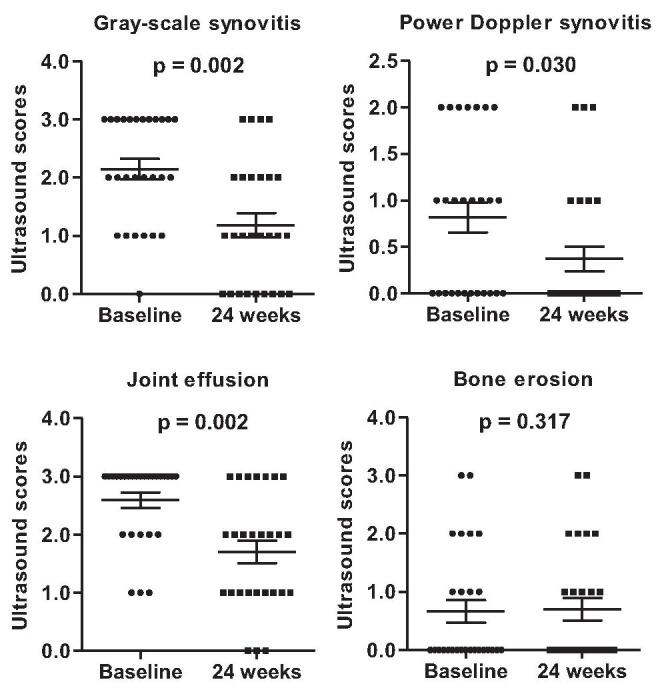
Comparison of ultrasound findings between baseline and 24 wk after baricitinib treatment.

Differences in ultrasound findings between baseline and 24 weeks after baricitinib are illustrated in Figure 2. Baricitinib for 24 weeks induced significant improvement of gray-scale synovitis, power Doppler synovitis, and joint effusion compared to baseline (2.00 [IQR 1.00–3.00] vs 1.00 [IQR 0.00–2.00], *P* = .002; 1.00 [IQR 0.00–2.00] vs 0.00 [IQR 0.00–1.00], *P* = .030; 3.00 [IQR 2.00–3.00] vs 2.00 [IQR 1.0–3.0], *P* = .002, respectively). In addition, bone erosion score was not different with baricitinib treatment for 24 weeks (*P* = .317).

**Figure 3:**
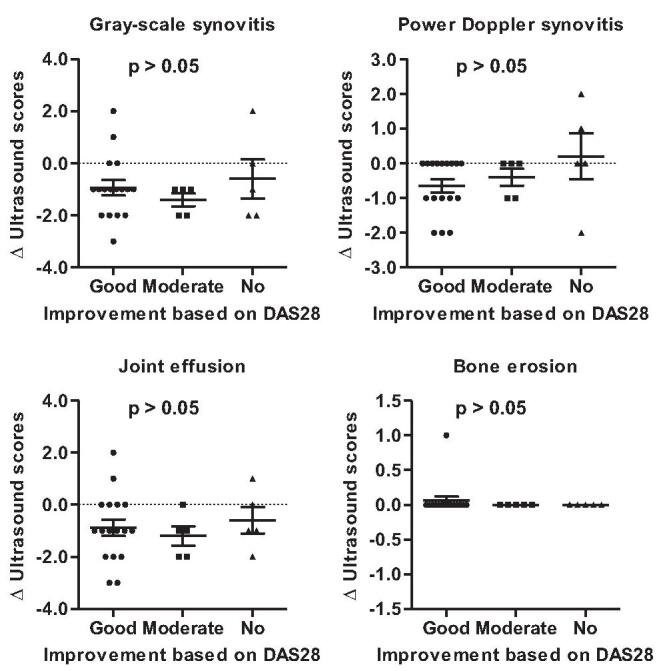
Comparison of changes in ultrasound findings based on DAS28-ESR improvement. DAS28-ESR = disease activity score for 28-joint count with erythrocyte sediment rate.

In analysis of the correlation between disease activity composites and ultrasound findings, multivariate regression analysis showed that power Doppler score was related to DAS28-ESR (β = 0.590, *P* = .044), but not SDAI and CDAI (Table 3). The relationship between disease activity composites and gray-scale synovitis was insufficient. Changes in ultrasound findings were compared based on DAS28-ESR improvement (Fig. 3). There were no significant changes in gray-scale synovitis, power Doppler synovitis, joint effusion, and bone erosion scores among patients with good, moderate, and no improvement (*P* > .05 for all).

**Table 3:** Correlation between changes of DAS28-ESR and synovitis scores for 24 wk.

	Changes of synovitis scores	
	ΔGray-scale score	ΔPower Doppler score
ΔDAS28-ESR		
Univariate analysis	β = 0.014, *P* = .954	β = 0.523, *P* = .018
Multivariate analysis^∗^	β = 0.043, *P* = .850	β = 0.590, *P* = .044
ΔSDAI		
Univariate analysis	β = 0.227, *P* = .255	β = 0.412, *P* = .033
Multivariate analysis^∗^	β = 0.324, *P* = .124	β = 0.558, *P* = .078
ΔCDAI		
Univariate analysis	β = 0.220, *P* = .271	β = 0.406, *P* = .036
Multivariate analysis^∗^	β = 0.308, *P* = .152	β = 0.539, *P* = .095

## 4 Discussion

The synovial immunologic and inflammatory response of RA is tightly controlled by a variety of cellular and humoral process.^[1]^ The JAK-signal transducer and activator of transcription (STAT) signaling pathway plays a role in mediating the function of inflammatory cytokines and growth factors associated with synovial inflammation and bone destruction in RA.^[1,17]^ Tight regulation of the JAK signaling pathway has emerged as a crucial therapeutic strategy in RA. Four phase 3, randomized, double-blind, multicenter studies demonstrated that baricitinib markedly improved clinical symptoms and signs in RA.^[8,9,10,11]^ However, there is insufficient efficacy data for ultrasound-based synovial inflammation using gray-scale and power Doppler synovitis for bone damage after treatment with baricitinib. This study showed that baricitinib treatment for 24 weeks significantly improved ultrasound-detected joint effusion, gray-scale, and power Doppler synovitis and inhibits progression of bone erosion in patients with RA.

Synovial tissue and fibroblasts play a crucial role in the inflammatory response in RA and further induce destructive cartilage and bone damages. Therefore, synovial tissue and fibroblasts are potential therapeutic targets in RA. Experimental evidence showed that JAK-STAT proteins are highly expressed in synovial fibroblasts and tissues.^[18,19]^ Tofacitinib, an oral JAK inhibitor that selectively inhibits JAK1 and JAK3 with less selectivity against JAK2, was approved for management of RA.^[20]^ JAK inhibition with tofacitinib suppressed the inflammatory response of rats with adjuvant-induced arthritis, illustrating decreased infiltration of synovial sub-lining and lining layers.^[21]^ Another study showed that increased synovial lining hyperplasia in a rabbit model of chronic antigen induced arthritis was significantly inhibited by tofacitinib.^[22]^ Changes in musculoskeletal images after treatment with tofacitinib were investigated using ultrasound. In a phase 4 study using ultrasound to assess treatment response in patients with RA who took tofacitinib 5 mg bid, gray-scale synovial hypertrophy and synovitis with power Doppler signal significantly improved at 12 weeks of tofacitinib treatment (https://clinicaltrials.gov/ct2/show/NCT02321930). Another study revealed that ultrasound-based score after 12 weeks of tofacitinib treatment was closely related to clinical and laboratory measures of disease activity.^[23]^

There is some experimental evidence regarding the therapeutic effect of baricitinib on synovial inflammation. Interferon-γ-induced gliostatin production was significantly suppressed by treatment with baricitinib in human fibroblast-like synoviocytes.^[24]^ In an in vitro experiment about the effect of different JAK inhibitors on proliferation of RA synoviocytes, tofacitinib, baricitinib, and peficitinib markedly blocked proliferation of synoviocytes stimulated by interleukin-1β, but not filgotinib.^[25]^ Torikai et al showed that 3 months of baricitinib improved clinical disease activity based on CDAI and also significantly decreased gray-scale and power Doppler scores at 1 and 3 months compared to baseline.^[14]^ Consistently, our study also confirmed that baricitinib is an effective therapeutic strategy for regulation of synovial inflammation in RA. This was supported by improved DAS28-ESR at 3 and 6 months compared to baseline and markedly decreased ultrasound-based gray-scale and power Doppler scores. Interestingly, we found no changes in bone erosion score for 6 months after treatment with baricitinib. This result implicates that baricitinib suppresses deterioration of bone erosion in RA.

Power Doppler in ultrasound is an imaging tool to assess active synovial inflammation by detecting increased microvascular blood in affected joints. Furthermore, it is being used more frequently to evaluate and monitor disease activity in RA.^[26]^ There are some debates regarding the association between power Doppler intensity and clinical disease activity composites. On ultrasound evaluation of 50 patients with RA in clinical remission, there was an association between power Doppler and CDAI (*P* = .005), but not DAS28-ESR (*P* = .11).^[27]^ In contrast, power Doppler was not associated with disease activity indexes such as DAS28-ESR, CDAI, and SDAI.^[28]^ In this study, we observed that changes of power Doppler signal between baseline and 24 weeks were related to changes of DAS28-ESR, but not SDAI and CDAI. This implicates that power Doppler signal based on ultrasound might reflect disease activity relatively well in RA.

Cumulative evidence suggests that the JAK-STAT pathway is involved in bone metabolism.^[17]^ JAK family proteins consisting of JAK1, JAK2, JAK3, and tyrosine kinase 2 induce production of pro-inflammatory cytokines through activation of STAT proteins.^[29]^ Some researchers investigated whether the JAK signal pathway plays a crucial role in osteoclastogenesis in RA-like inflammatory arthritis. Tofacitinib did not show an inhibitory effect on RANKL-induced osteoclast formation or differentiation in inflammatory arthritis animal models.^[30,31]^ In contrast, tofacitinib treatment markedly prevented bone erosion in Wistar rats with adjuvant-induced arthritis compared to rats with arthritis not receiving tofacitinib.^[21]^ Conversely, the role of baricitinib in bone remodeling is not clearly identified in RA. Murakami et al demonstrated that baricitinib inhibited osteoclast differentiation from bone marrow cells under stimulation with 1,25-dihydroxyvitamin D_3_ and prostaglandin E_2_ and suppressed RANKL expression in osteoblastic cells.^[32]^ They proposed that inhibition of JAK1 and JAK2 is responsible for induction of RANKL, which blocks osteoclast formation in inflammatory arthritis. In the RA-BUILD study, tofacitinib treatment for 24 weeks reduced radiographic progression of structural joint damage compared to a placebo group.^[9]^ Consistently, our study showed that there was no further increase in bone erosion in affected joints after baricitinib treatment for 24 weeks. This result suggests that baricitinib can prevent and reduce bony structural damage in RA.

This study involves some limitations. First, ultrasound-detected findings were evaluated for only 1 or 2 joints showing the most severe abnormalities in each patient. Evaluation of these joints assessed using ultrasound does not fully reflect the overall inflammatory changes of other affected joints. Second, the study population may be too small to prove relevant changes in ultrasound and clinical findings. However, prior power calculation revealed that 27 painful joints in 20 patients would be appropriate to confirm the changes of ultrasound-detected abnormalities. The statistical power (1-β) was set to 0.8. Third, the absence of a control group treated with only csDMARDs without baricitinib may be a limitation to determine the superiority of baricitinib to ultrasound findings.

In conclusion, our data provided evidence that treatment with baricitinib 4 mg for 24 weeks showed a beneficial effect on synovial inflammation and bone erosion in patients with RA with inadequate response to csDMARDs or bDMARDs. Long-term follow-up study is needed to confirm if the therapeutic effect of baricitinib on relieving ultrasound findings remains.

## Author contributions

**Conceptualization:** Seong-Kyu Kim.

**Data curation:** Seong-Kyu Kim, Ui Hong Jung, Ji-Won Kim, Jung-Yoon Choe.

**Investigation:** Seong-Kyu Kim, Ui Hong Jung, Ji-Won Kim, Jung-Yoon Choe.

**Methodology:** Seong-Kyu Kim, Ui Hong Jung, Ji-Won Kim, Jung-Yoon Choe.

**Supervision:** Seong-Kyu Kim.

**Writing – original draft:** Seong-Kyu Kim.

**Writing – review & editing:** Seong-Kyu Kim.

## Supplementary Material

Supplemental Digital Content
