# Supplementary material for: The beneficial effect of baricitinib on ultrasound-detected synovial inflammation and bone damage in rheumatoid arthritis: Preliminarily data from single center-based observational study for 24 weeks
Source: Medicine (Baltimore). 2021 Jul 30;100(30):e26739. doi: 10.1097/MD.0000000000026739 (PMC8322478; doi:10.1097/MD.0000000000026739)
Supplement: Supplemental Digital Content [file medi-100-e26739-s001.doc]

Supplementary table 1. Scoring system for ultrasound abnormalities

| Abnormalities | Definition |
| --- | --- |
| Synovitis |  |
| Joint effusion | Grade 0: no effusion  Grade 1: minimal amount of joint effusion  Grade 2: moderate amount of joint effusion  Grade 3: extensive amount of joint effusion |
| GS Synovitis | Grade 0: no synovial thickening  Grade 1: minimal synovial thickening (filling the angle between the periarticular bones, without bulging over the line linking tops of the bones)  Grade 2: synovial thickening bulging over the line linking tops of the periarticular bones but without extension along the bone diaphysis  Grade 3: synovial thickening bulging over the line linking tops of the periarticular bones and with extension to at least one of the bone diaphyses |
| PD synovitis | Grade 0: no flow in the synovium  Grade 1: single vessel signals  Grade 2: confluent vessel signals in less than half of the area of the synovium  Grade 3: vessel signals in more than half of the area of the synovium |
| Bone erosion |  |
|  | Grade 0: regular bone surface  Grade 1: irregularity of the bone surface without formation of a defect seen in two planes  Grade 2: formation of a defect in the surface of the bone seen in 2 planes  Grade 3: bone defect creating extensive bone destruction |

Reference: Szkudlarek M, et al. Arthritis Rheum 2003;48:955-62.
